# Risk factors and consequences of undiagnosed cesarean scar pregnancy: a cohort study in China

**DOI:** 10.1186/s12884-019-2523-0

**Published:** 2019-10-26

**Authors:** Ri-hua Xie, Xiaoyan Guo, Meng Li, Yan Liao, Laura Gaudet, Mark Walker, Huizhong Lei, Shi Wu Wen

**Affiliations:** 1 Department of Nursing, General Practice Center, Nanhai Hospital, Southern Medical University, Foshan, Guangdong China; 2 Department of Obstetrics and Gynecology, the Sixth Affiliated Hospital of Guangzhou Medical University, Qingyuan, Guangdong China; 3 Department of Obstetrics, Nanhai Hospital, Southern Medical University, Foshan, Guangdong China; 4 Ottawa Hospital Research Institute, Ottawa, ON Canada; 5 OMNI Research Group, Department of Obstetrics and Gynecology, University of Ottawa, 501 Smyth Road, Box 241, Ottawa, ON K1H 8L6 Canada; 6 BORN (Better Outcome Registry Network) Ontario, Children’s Hospital of Eastern Ontario, Ottawa, ON Canada; 7 School of Epidemiology and Public Health, University of Ottawa Faculty of Medicine, Ottawa, ON Canada

**Keywords:** Cesarean scar pregnancy, Undiagnosed, Risk factors, Adverse outcomes

## Abstract

**Background:**

The historically high cesarean section rate and the recent change in second-child policy could increase the risk of cesarean scar pregnancy (CSP) in China. This study aims to assess risk factors and consequences of undiagnosed CSP in China.

**Methods:**

We conducted a retrospective cohort study between January 2013 and December 2017 in Qingyuan, Guangdong, China. Independent risk factors for undiagnosed CSP at the first contact with healthcare providers were assessed by log binomial regression analysis. Occurrence of serious complications was compared between undiagnosed and diagnosed CSP cases.

**Results:**

A total of 195 women with CSP were included in the analysis. Of them, 81 (41.5%) women were undiagnosed at the first contact with healthcare providers. Women initially cared in primary or secondary hospitals were at increased risk for undiagnosed CSP: adjusted relative risks (95% confidence intervals) were 3.28 (2.06, 5.22) and 1.91 (1.16, 3.13), respectively, compared with women initially cared in the tertiary hospital. Undiagnosed CSP cases had higher incidences in serious complications (11 versus 0) and post-surgery anemia (23 (28.4%) versus 8 (7.0%)), stayed longer in hospital, and cost higher than diagnosed CSP cases.

**Conclusions:**

Initial care provided at primary or secondary maternity care facilities is an important risk factor for undiagnosed CSP, with serious consequences to the affected women.

## Background

Cesarean scar pregnancy (CSP) is a special type of ectopic pregnancy, in which the fertilized egg and trophoblast cells are implanted after cesarean section [1–4]. The incidence of CSP varies greatly from 1/1800 to 1/7500 pregnancies [4–6]. This is related to the high cesarean section rate in the past decades [2]. The recent relaxation of the second-child policy might increase additional risk of CSP in reproductive age women with a history of cesarean section in China [7].

Clinical symptoms of CSP are not typical, thus, diagnosing accurately based on irregular menstrual period, vaginal bleeding, and urine human chorionic gonadotropin (HCG) positive alone is difficult. If CSP is not diagnosed timely, the pregnancy will continue and lead to serious complications such as placenta accreta, placenta previa, uterine rupture, and heavy bleeding during surgery [1, 3, 4]. With a combination of cesarean section history and accurate ultrasound and/or MRI examination, a timely and correct diagnosis can reduce occurrence of these complications [1, 3, 4].

Most previous studies on CSP were case reports or case series with no formal assessment of risk factors for undiagnosed CSP [1, 5, 6, 8–21]. Therefore, the present retrospective cohort study aims to explore the risk factors of undiagnosed CSP at the first contact with healthcare providers and to assess adverse outcomes of undiagnosed CSP cases.

## Methods

### Study design and study setting

In this cohort study, we used data collected for routine quality assurance purposes, thus, the Research Ethics Board of The Sixth Affiliated Hospital of Guangzhou Medical University concluded that an ethical approval was not required and no consent from patients was needed.

The study was conducted in Qingyuan region that is a prefecture-level region in northern part of Guangdong province in China [22]. There are 91 qualified primary maternity care facilities and 15 qualified secondary maternity care facilities. The primary care facilities are mostly township hospitals with 1–2 attending physicians who had general medical training and the secondary care facilities are mostly county hospitals with 3–4 attending physicians who had some specialized training in maternity care. There are general ultrasound machines in both township and county hospitals. However, there are no obstetric specific ultrasound machines in these hospitals. Only non-complicated surgeries such as therapeutic abortion and elective cesarean section can be performed in these two-level hospitals. The Sixth Affiliated Hospital of Guangzhou Medical University, where data on CSP cases for this study were collected, is the only tertiary care hospital in the region, with an experienced maternity care team. In 2017, 18 attending physicians in this team had complete specialty training in maternity care. This maternity care team is also provided with facilities for diagnosis and treatment of high-risk pregnancy, including monitors, 4 D ultrasound machines, color ultrasound machines, transvaginal ultrasound machines, and supported by hospital’s anesthesia, surgery, and intensive care teams. Although all three levels of maternity care facilities in Qingyuan provide healthcare to low-risk pregnancies, women with an elevated risk are referred to higher level of hospitals, depending on clinical conditions, either to secondary hospitals (middle risk) or to the tertiary hospital (high risk). For high-risk pregnancies with no emergency, the referral time is normally 4 to 7 days, and for the high-risk pregnancies with emergency, it is 0.5 to 4 h (depending on the distance and the means of transportation). All CSP women were referred to the tertiary hospital, and few CSP women in the region sought treatment outside (for example, in 2017 official records, no CSP case was treated in any hospital outside of Qingyuan).

### Study sample

All CSP cases in Qingyuan from January 2013 to December 2017 were included in this study. Demographic and clinical data were retrieved from the medical charts by trained personnel.

### Outcome measures

The primary outcome of this study was undiagnosed CSP at the first contact with healthcare providers. All CSP cases were eventually diagnosed accurately at the tertiary hospital using data from multiple sources, including history of cesarean section, clinical manifestation, ultrasound scan, Doppler probe, and postoperative pathological examination. However, many CSP cases were undiagnosed at the first contact with healthcare providers.

Secondary outcomes included serious post-surgical complications, post-surgical anemia, length of hospital stay, and hospital cost. Serious complications in this study included placenta accreta, placenta previa, uterine rupture, heavy bleeding during surgery, and “near miss”. Placenta accrete, placenta previa, and uterine rupture were based on clinical diagnosis, and heavy bleeding during surgery was recorded by on-duty attending physicians according to their judgement of the patient’s condition. “Near miss” is a serious adverse event that a pregnant woman comes close to death, but from which she survives. Post-surgical anemia is defined as hemoglobin level of 6–9 g/100 ml.

### Risk factors of undiagnosed CSP

Risk factors included maternal age, parity, rural resident, gestational age, and type of health care facility at the first contact with healthcare providers.

### Statistical analysis

We first described baseline characteristics of study participants. We then analyzed risk factors of undiagnosed CSP. A log binomial model was used to estimate independent effect of risk factors, with undiagnosed CSP as outcome measure, and relative risk (RR) and 95% confidence interval (CI) as effect measure. Full model with all risk factors included in the regression analysis was performed. There were two levels of independent variables: the type of health care facilities measured at the hospital level and the remaining variables measured at the patient level. We thus used multi-level model to run log binomial regression. We also compared secondary outcomes between undiagnosed CSP cases and diagnosed CSP cases at the first contact with healthcare providers. Chi-square test was used to compare categorical outcomes and t-test was used to compare continuously distributed outcomes. Finally, we explored the main reasons for undiagnosed CSP at the first contact of healthcare providers.

## Results

### Occurrence of undiagnosed CSP at the first contact with healthcare providers

There were 324,335 births in Qingyuan during the study period, of them, 195 CSP cases were identified, yielding a rate of 60 CSP cases per 100,000 births. Of the 195 CSP cases, 81 (41.5%) were undiagnosed at the first contact with healthcare providers.

### Characteristics of CSP cases

Table 1 shows characteristics of CSP cases. Average age of these women was 32 years and average timing of the first contact with healthcare providers was 7.6 gestational weeks, with > 60% of them initially seeking care at primary (level 1) or secondary (level 2) health care facilities (Table 1).

**Table 1:** Characteristics of CSP cases, Qingyuan, Guangdong, China, 2013 to 2017

Characteristics	
Age in year, Mean+_SD, Medium, (range)	31.9 ± 5.4, 32, 19–44
Parity (n, %)	
1	106 (54.36)
> = 2	89 (45.64)
Rural residence (n, %)	
Yes	120 (61.540029
No	75 (38.46)
Gestational age at the first contact with healthcare provider, s, MeanSD, Medium, (range)	7.6 ± 2.2, 7.1, (4.57, 21.7)
Type of initial health care (n, %)	
Primary^a^	56 (28.72)
Secondary	61 (32.28)
Tertiary	78 (40.00)
Undiagnosed CSP (n, %)	
Yes	81 (41.54)
No	114 (58.46)

^a^Including one patient self-treated using medications, without seeking care from any healthcare provider

### Risk factors of undiagnosed CSP at the first contact with healthcare providers

Table 2 presents risk factors of undiagnosed CSP at initial contact with a healthcare provider. Initial care provided at primary and secondary health care facilities was the only independent risk factor, which was significantly associated with an increased risk of undiagnosed CSP. Compared with those patients initially cared at the tertiary hospital, adjusted RRs (95% CIs) were 3.28 (2.06, 5.22) and 1.91 (1.16, 3.13), respectively, for those who were initially cared at primary and secondary health care facilities. No statistically significant association with undiagnosed CSP was observed in other risk factors (Table 2).

**Table 2:** Risk factors of undiagnosed CSP at the first contact of healthcare providers, Qingyuan, Guangdong, China, 2013 to 2017

Determinants	Number of undiagnosed CSP	Rate of undiagnosed CSP (%)	Crude RR (95% CI)	Adjusted RR (95% CI)
Age				
< 30 (*n* = 68)	31	45.59	Reference	Reference
> 30 (*n* = 127)	50	39.37	0.86 (0.62, 1.21)	1.02 (0.64, 1.64)
Parity				
1 (*n* = 106)	45	42.45	Reference	Reference
> =2 (*n* = 89)	36	40.45	0.96 (0.7, 1.31)	0.87 (0.70, 1.09)
Rural residence				
Yes (*n* = 120)	50	41.33	1.01 (0.8, 1.26)	0.97 (0.73, 1.29)
No (*n* = 75)	31	41.33	Reference	Reference
Gestational age in week at initial contact with healthcare providers				
< 6 weeks (*n* = 41)	12	29.27	Reference	Reference
6-7 weeks (*n* = 92)	33	35.87	1.09 (0.87, 1.38)	1.26 (0.86, 1.85)
> = 7 weeks (*n* = 62)	36	58.06	1.59 (1.15, 2.19)	1.82 (0.98, 3.37)
Type of initial health care facilities				
Primary (*n* = 56)	41	73.21	3.81 (2.35, 6.16)	3.28 (2.06, 5.22)
Secondary (*n* = 61)	25	40.98	1.72 (1.21, 2.45)	1.91 (1.16, 3.13)
Tertiary (*n* = 78)	15	19.23	Reference	Reference

### Comparison of secondary outcomes between undiagnosed and diagnosed CSP cases

Table 3 shows outcomes between undiagnosed and diagnosed CSP cases. Undiagnosed CSP cases had higher rates of anemia, longer hospital stays, and higher cost than diagnosed CSP cases (Table 3). Eleven undiagnosed CSP cases developed serious complications such as placenta accreta, placenta previa, uterine rupture, heavy bleeding during surgery, or were given a diagnosis of “near miss” (Table 3).

**Table 3:** Comparison of outcomes between undiagnosed and diagnosed CSP at the first contact with healthcare providers, Qingyuan, Guangdong, China, 2013 to 2017^a^

Outcomes	Undiagnosed (*N* = 81)	Diagnosed (*N* = 114)	*P* Value
Serious complications (n, %)^a^	11 (13.6)	0 (0.0)	< 0.001
Anemia (n, %)^b^	23 (28.4)	8 (7.0)	< 0.01
Length of hospital stay (in days; Mean, SD)^c^	5.5 ± 3.2	4.6 ± 2.0	0.03
Hospital cost (in RMB Yuan; Mean, SD)^d^	8626.2 ± 5995.1	6199.0 ± 3859.7	< 0.01

^a^Occurrence of any of the following condition: placenta accrete, placenta previa, uterine rupture, heavy bleeding, and near miss; difference between the two groups was compared by Fisher exact test

^b^Difference between the two groups was compared by chi-square test

^c^Difference between the two groups was compared by t-test

^d^One US dollar was about 6.5 RMB during the study period; Difference between the two groups was compared by t-test

### Actual diagnosis for undiagnosed CSP cases at the first contact with healthcare providers

Table 4 displays the diagnosis of cases that initially failed to be recognized as CSP. Most of these cases were initially diagnosed as normal early pregnancy followed by abortions (Table 4).

**Table 4:** Initial diagnosis of undiagnosed CSP cases at the first contact of healthcare providers, Qingyuan, Guangdong, China, 2013 to 2017

Initial diagnosis	Number of undiagnosed cases (*N* = 81)	As a percent of all undiagnosed cases (%)
Normal early pregnancy	40	49.4
Abortions	27	33.3
Other ectopic pregnancy	3	3.7
Others	11	13.6

## Discussion

### Main findings

Our study found that a large proportion of CSP cases (41.5%) were undiagnosed as CSP but were diagnosed as normal pregnancies or abortions at the first contact with healthcare providers. The only significant risk factor for undiagnosed CSP was the first point of care at a primary or secondary level hospital. Undiagnosed cases had higher rates of serious complications and post-surgery anemia, stayed longer in hospital, and had higher cost than diagnosed cases.

### Strengths and limitations

To our knowledge, this is the first study that explored risk factors of undiagnosed CSP at the first contact of healthcare providers and compared outcomes between undiagnosed CSP cases and diagnosed CSP cases [1, 5, 6, 8–21]. The study population included all CSP cases who were treated in the catchment area, therefore, there was no selection bias. This study is also one of the largest in the field [1, 5, 6, 8–21]. The final diagnosis of CSP was valid and solid, and the chance of misclassification at the final diagnosis was unlikely. We analyzed risk factors for undiagnosed CSP cases from both the patient and healthcare provider perspective, which helps to interpret the results and compare with previous studies.

The maternity care team of the only tertiary maternity care hospital in Qingyuan region is experienced in treating high-risk pregnancies, which ensured that no death occurred in the CSP cases during the study period. However, serious complications such as placenta accreta, placenta previa, uterine rupture, and heavy bleeding did occur in CSP cases who were undiagnosed at the first contact with healthcare providers, and two such cases were in “near miss”.

We used post-surgical anemia to serve as an indicator of the severity and complexity of the patient’s condition. Data on laboratory-diagnosed anemia from medical charts are reliable. In this study, more than 28% of women with undiagnosed CSP developed post-surgical anemia, while only 7% with diagnosed CSP. Moreover, undiagnosed CSP cases had longer hospital stays and higher cost than diagnosed cases, indicating that undiagnosed CSP not only affects the patient’s health, but also imposes a burden on the health care system. However, sample size of this study may be limited to assess smaller effects. Some factors such as gestational age at the first contact with healthcare providers may become significant if a larger study sample is available. The data did not have information on previous pregnancies such as quality and healing process of previous cesarean section, which prevented us from in-depth investigation of the causes for failure to a timely diagnosis of CSP.

### Interpretation

The rate of CSP in this study was in the high end among previous studies [4–6]. This is not a surprise given the historically high cesarean section rate [2] and the recent change in second-child policy in China [7]. Most previous studies in this field described clinical features of CSP cases only [1, 5, 6, 8–21], with no attempt to examine risk factors for undiagnosed CSP at initial contact with healthcare providers. Risk of CSP appears to be increased with the number of cesareans, history of dilatation and curettage, placental pathology, history of ectopic pregnancy, and use of assisted reproductive technology such as IVF [4]. Although the importance of timely diagnosis for CSP has been discussed in these studies [1, 5, 6, 8–21], risk factors for failure to make a timely diagnosis have not been assessed.

Risk factors for CSP are different from risk factors for undiagnosed CSP. Our study suggests that for diagnosis, risk factors from healthcare providers may be more important than those from patients, because patient’s characteristics (e.g. age, parity, rural residence, and gestational age) were not statistically significantly associated with diagnosis, while the level of health care facility was. We should emphasize that, in this study, undiagnosed CSP refers to the diagnosis at patient’s first contact with a healthcare provider - eventually all CSP cases were diagnosed accurately. Delaying in diagnosis contributes greatly to adverse outcomes. If a diagnosis of normal pregnancy is made at the initial visit, pregnancy may continue and lead to serious conditions such as placenta accreta or placenta previa. As in other aspects of health care and in other parts of China [23, 24], huge variations in terms of competence of health care providers and facilities in maternity care exist in Qingyuan. Primary care is mostly provided by township hospitals and secondary care is mostly provided by county hospitals. In these primary and secondary care facilities, medical workers, including both sonographers and physicians, often lack relevant CSP knowledge to make an accurate diagnosis [25]. Furthermore, these hospitals have no vaginal ultrasound or color Doppler and only have abdominal ultrasound. Abdominal assessment lacks resolution, particularly in early pregnancy and may not have an accurate diagnosis. It is, therefore, crucial to ensure that medical staff at these hospitals take carefully medical histories including cesarean section and vaginal bleeding and transfer patients with a suspected CSP to tertiary care centers for further assessment.

We searched Medline with key words of “Cesarean Scar Pregnancy AND Diagnosis”, and identified one case report that described a CSP that was initially diagnosed as another condition despite using ultrasound [21]. This report emphasizes the need for formal training of staff in all maternal care centers. Transvaginal ultrasound examination has advantages of being easy to operate, repeatable, and low cost, and should be the first choice. Diagnosis of CSP by vaginal ultrasonographic examination could be established as follows: 1) uterus is slightly enlarged, 2) there is no pregnancy in uterine cavity, 3) endometrial line is clearly visible, 4) a gestational sac with a york sac or crown length with or without heart rate, or complex mass with mixed echogenicity is located in the myometrium at the level of lower uterine segment, and is surrounded by visible blood flow, and 5) muscular layer between gestational sac and bladder is thin [3–6, 26–30]. Any pregnant woman with a history of cesarean section should be evaluated for CSP, using a thorough ultrasound assessment. For pregnant women with a history of cesarean section and vaginal bleeding, blind suction or curettage is dangerous.

## Conclusions

Initial care provided at primary or secondary maternity care facilities is an important risk factor for undiagnosed CSP, with serious consequences to the affected women.

High degree of awareness, detailed history, skillful ultrasound examination, and timely referral of suspected CSP patients to a tertiary care center are keys for accurate diagnosis.

## Data Availability

The data of this study are stored in secured servers of the institutions and are available from the corresponding authors on reasonable request.

## Abbreviations

CI: Confidence interval
CSP: Cesarean scar pregnancy
HCG: Human chorionic gonadotropin
IVF: In vitro fertilization
MRI: Magnetic resonance imaging
RR: Relative risk
